# Pre- and postfracture patient-reported outcomes for patients undergoing periprosthetic femoral fractures after primary total hip arthroplasty—An observational study

**DOI:** 10.1177/00368504251396075

**Published:** 2025-12-01

**Authors:** Marte Stine Stovner, Tina Strømdal Wik, Tone Gifstad

**Affiliations:** 1 Department of Orthopedic Surgery, Namsos Hospital, Namsos, Norway; 2 Norwegian University of Science and Technology, Trondheim, Norway; 3 Department of Orthopedic Surgery, St. Olavs Hospital, Trondheim, Norway

**Keywords:** Arthroplasty, periprosthetic fracture, PROMs, THA, femoral stem

## Abstract

**Objective:**

Periprosthetic femoral fracture has become the most common reason for reoperation after total hip arthroplasty (THA). The aim of this study was to evaluate patient-reported outcomes (PROMs) after undergoing surgery due to a periprosthetic femoral fracture.

**Methods:**

Patients registered with a periprosthetic femoral fracture in the surgical theater protocols and in advance included in an institutional quality register were included in the study. PROMs were compared for each patient before primary THA, at 3 and 12 months follow-up and at the endpoint after fracture surgery. The PROMs investigated were numeric rating scale pain, Harris Hip Score (HHS), Hip disability and Osteoarthritis Outcome Score—Physical Function Short-form (HOOS-PS) and Forgotten Joint Score.

**Results:**

A total of 27 patients were included in the study. Pain level after periprosthetic fracture surgery were comparable to the pain level registered at 3 and 12 months after THA. The same were found for HHS and HOOS-PS. In addition, both pain scores, HHS and HOOS-PS were significantly improved after fracture surgery compared to before the primary THA.

**Conclusions:**

Pain and function scores after periprosthetic surgery were comparable to the findings at 3 and 12 months follow-up after primary THA surgery. Despite a limited number of patients, the present study report unique results including PROMs both pre- and post-THA and after the periprosthetic femoral fracture.

## Introduction

The incidence of periprosthetic femoral fractures for primary total hip arthroplasty (THA) has been reported from 1% to 4%^1–3^ and is now the most common reason for reoperation after hip arthroplasty in Norway.^4^ Factors such as female gender, osteoporosis, rheumatoid arthritis, cementless implants, malposition of the implants, osteolysis or loosening, and higher ASA-classification are all associated with increasing risk of periprosthetic femoral fractures.^5–8^ Undergoing a periprosthetic femoral fracture is associated with functional limitations and increased overall mortality.^9^ Functional outcomes after revision for periprosthetic fractures are scarcely studied and reported results do not include prefracture data. Moreta et al.^10^ reported in 2015 the functional and radiological outcomes for 43 patients with Vancouver B2 and B3 fractures in a retrospective study. The population studied were patients receiving a THA due to osteoarthritis or a proximal femoral fracture and they concluded that half of the patients did not return to their previous ambulatory level.^10^ Mulay et al.^11^ examined 24 patients six months postoperatively after Vancouver B2 and B3 fractures, and found that average Harris hip score (HHS) was 69 at this follow-up. The patient populations in these studies were not only periprosthetic fractures after elective primary THA, but they also included periprosthetic fractures after THA due to hip fractures or revision THA. In the present study, we aimed for a more homogenous clinical starting point before fracture and included only patients who had been included an institutional quality register before they underwent surgery with elective primary THA. The aim of this study was to evaluate patient-reported outcome measures (PROMs) for patients with periprosthetic femoral fracture after elective primary THA surgery. The patient population was followed prospectively from before their primary THA to postoperative follow-ups the first year after surgery, and further to postfracture results by using data from an institutional quality register and a follow-up evaluation.

## Patients and methods

All patients undergoing surgery due to a periprosthetic femoral fracture between January 2010 and December 2020 were identified by scanning the surgical theater protocols, detecting all patients undergoing surgery due to a periprosthetic femoral fracture at our department. The list of patients was cross-checked with patients registered with periprosthetic fractures in the national arthroplasty register. Patients with preoperative data in the institutional quality register when undergoing primary THA were found eligible to be inlcluded in the study, and invited to enroll in the study and undergo a clinical follow-up evaluation. Patients undergoing periprosthetic femoral fracture after THA due to a proximal femoral fracture or revision THA, and total knee arthroplasty related fractures were excluded. Patients deceased before the follow-up evaluation were also excluded. Patient demographics such as the American Society of Anesthesiology (ASA) score^12^ and body mass index at primary and fracture surgery were collected from the patient journals. Information regarding postoperative infections after fracture surgery was also noted. The study was conducted in accordance with the Helsinki Declaration of 1975 as revised in 2024. The reporting of this study conforms to STROBE guidelines.^13^ All patient-related data were deidentified.

### PROMs and clinical evaluation

### Index surgery evaluation

PROMs were prospectively collected in the institutional register before the index surgery and at 3 and 12 months follow-up. The numeric rating scale (NRS) for pain at rest and pain during activity were used as a rating-tool regarding pain.^14,15^ Patients were asked to rate their level of pain on an 11 point scale where 0 equals no pain and 10 the worst pain imaginable. The HHS^16,17^ and the Hip disability and Osteoarthritis Outcome Score—Physical Function Short-form (HOOS-PS)^18^ were used to evaluate the function. The Harris hips score rates from 0 to 100, and according to the interpretation of HHS, 70–79 points is a fair result, and 80–89 points is a good result.^17,19^ The HOOS-PS was scored from no difficulty (0) to extreme difficulty (100).

### Postfracture evaluation

Patients included in the study were invited to a clinical postfracture follow-up evaluation. The patients answered the same PROMs as during the index surgery evaluation. In addition the Forgotten Joint Score (FJS), a validated questionnaire used to evaluate function,^20^ was collected. Limb length discrepancy (LLD) Trendelenburg gait and type of fracture treatment was registered.

### Radiological evaluation

The periprosthetic femoral fractures were classified based on available X-rays using the Vancouver classification system.^21,22^

### Statistics

PROMs at the different follow-ups were displayed by plots with error bars indicating 95% confidence intervals (CI). Due to a limited number of patients, the number of statistical analyses was kept to a minimum. A *p*-value < .05 was considered significant when comparing PROMs before THA and at the final follow-up evaluation with the nonparametric Wilcoxon test. All statistical analyses were conducted with IBM SPSS Statistics 28.

## Results

**Figure 1.**
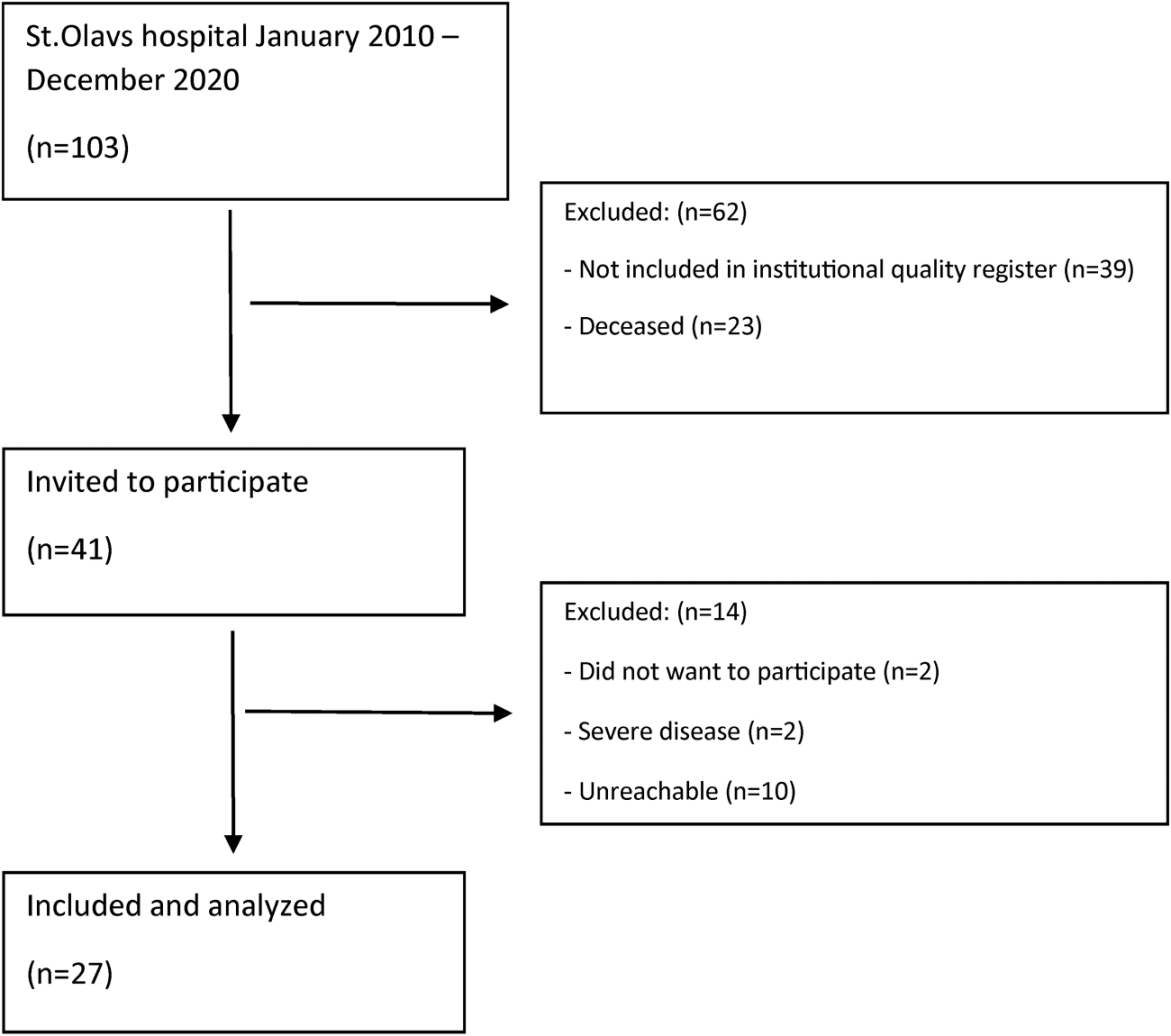
Flow chart—patients undergoing surgery due to a periprosthetic femoral fracture.

A total of 103 patients underwent surgery due to periprosthetic femoral fractures during 2010 to 2020. A total of 41 patients were found eligible and invited to participate in the study. Twenty-seven patients were consented and underwent clinical evaluation and registration of postfracture PROMs (Figure 1). Approximately 330 patients receiving primary hip arthroplasty were included in the institutional registry per year during the time-period studied.

Patient demographics are presented in Table 1. Four of the periprosthetic femoral fractures were intraoperative fractures detected on postoperative x-ray and four fractures were diagnosed within the first four weeks after surgery without a new trauma. All of these eight patients had an uncemented stem (six fully coated and two proximally coated).

**Table 1.** Patient demographics at the time of fracture surgery.

Mean age (years)	71 (54–80)
Gender (women/men)	21 women/6 men
Mean BMI	28 (18–43)
Mean time fracture to follow-up (years)	4 (1–10)

**Figure 2.**
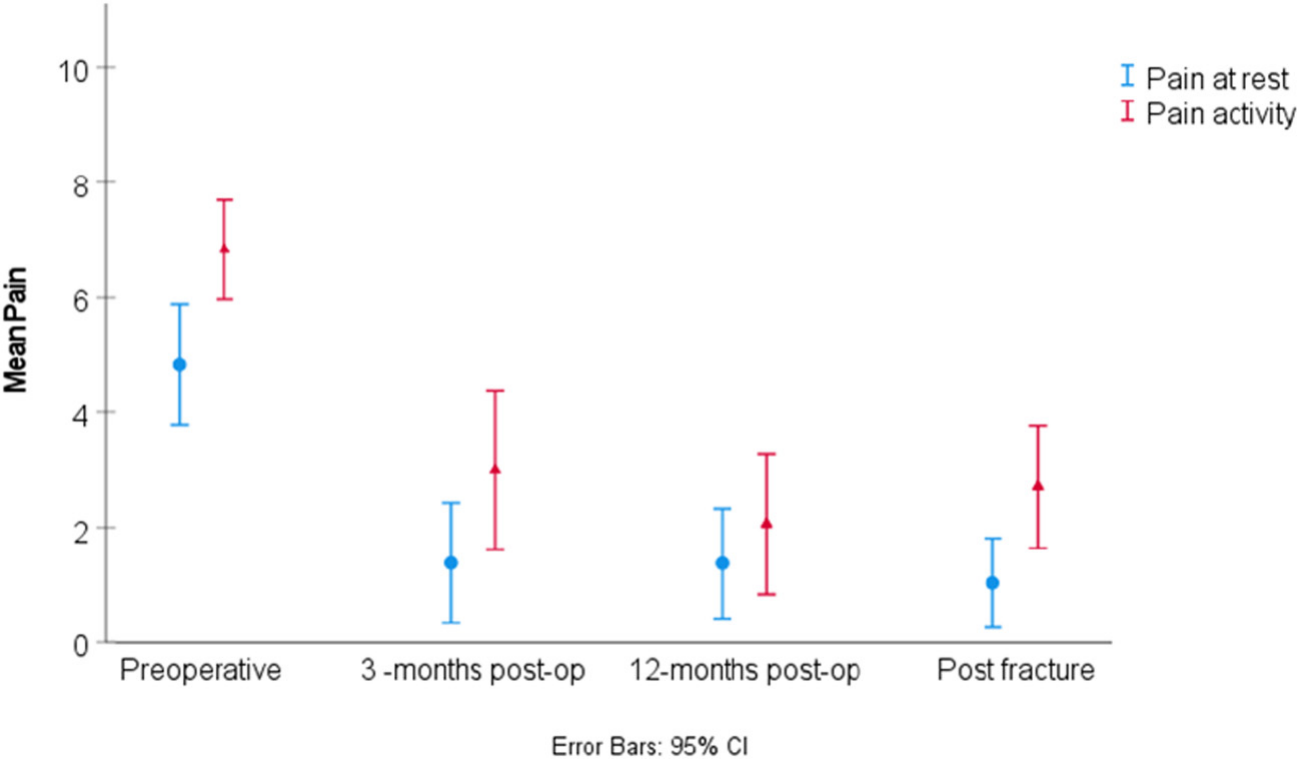
Pain at rest and during activity at various time-points.

**Figure 3.**
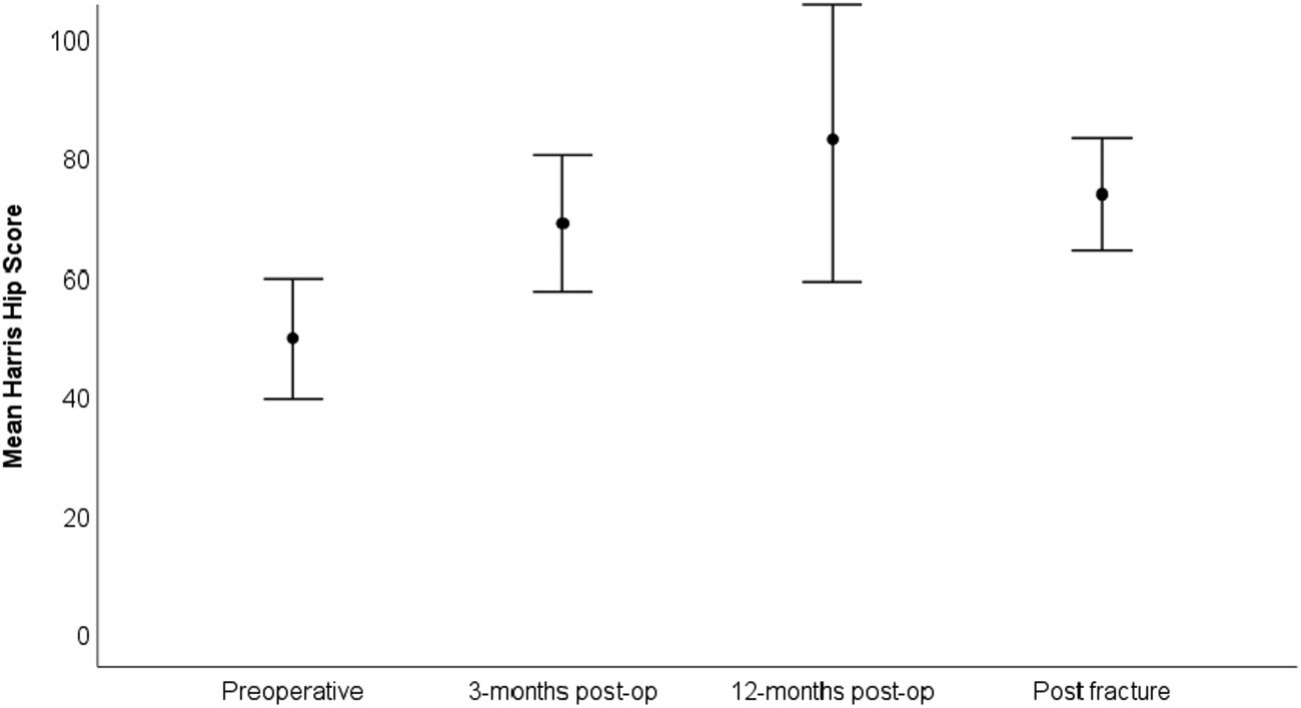
Harris hip score at various time-points.

**Figure 4.**
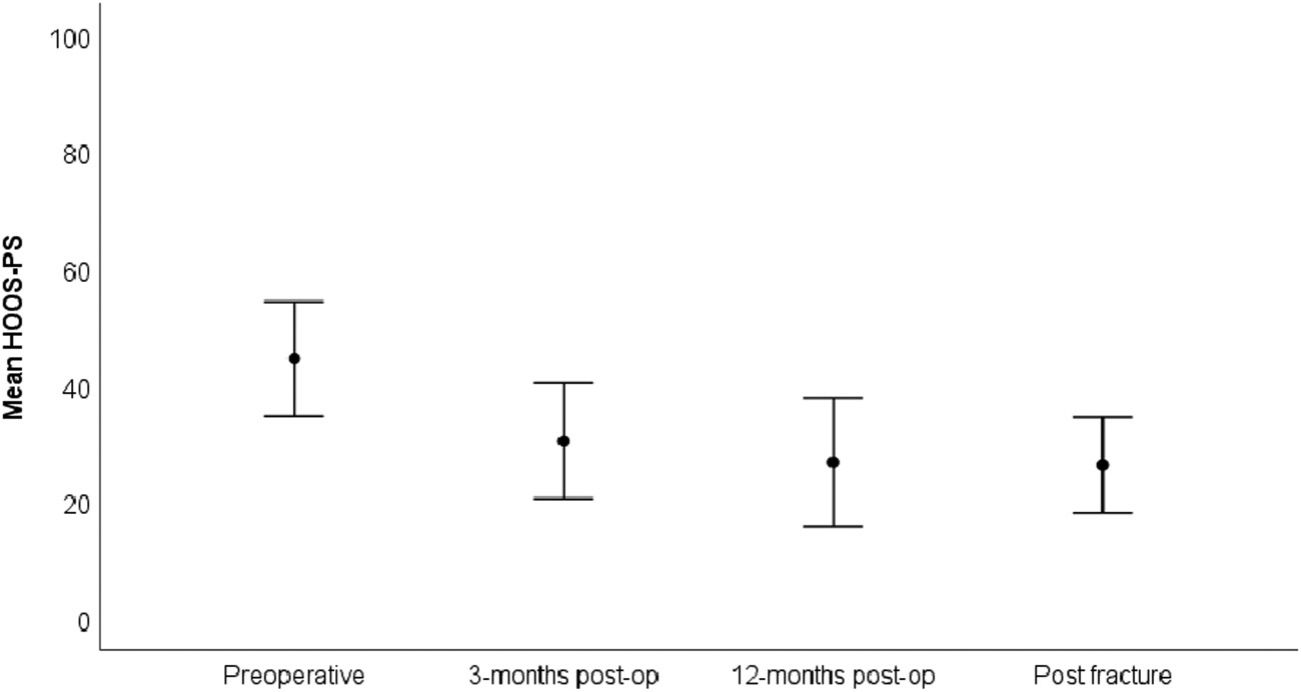
HOOS-PS at various time-points.

Mean pain level was higher before primary THA compared with all subsequent time-points (Figure 2). Pain scores after undergoing surgery for a periprosthetic femoral fracture were comparable to the values registered 3 and 12 months after primary THA (Figure 2). The HHS at 3 months and 12 months after primary THA was 69 and 83 respectively, and 74 at the postfracture follow-up (range 26 to 100) (Figure 3). HOOS-PS at 3 months and 12 months was 30 and 27 respectively, and 26 at the postfracture follow-up (range 0–82) (Figure 4). Significant improvements were found for all PROMs when comparing values before THA and at the final postfracture follow-up evaluation using the Wilcoxon test (pain at rest (*p* < .001), pain during activity (*p* < .001), HHS (*p* = .004) and HOOS-PS (*p* = .002)). Mean FJS at postfracture follow-up was 58 (0–100), SD 41 (Table 2).

HOOS-PS: Hip disability and Osteoarthritis Outcome Score—Physical Function Short-form.

**Table 2.** Vancouver classification and patient distribution.

Vancouver	Frequency (number of patients)	Time THA* to fracture (months)	Age (years)	Time fracture to follow-up (months)	Mean FJS** (range)
A	5	1 (0–5)	72 (61–72)	47 (9–114)	25 (0–100)
B1	2	20 (11–30)	73 (66–80)	61 (33–89)	100
B2	15	14 (0–71)	70 (54–78)	52 (9–126)	63 (0–100)
B3	1	103	74	20	85
C	4	21 (7–34)	73 (63–79)	60 (37–81)	51 (8–98)

* Total hip arthroplasty.

** Forgotten Joint Score.

The most frequent fracture pattern was Vancouver B2 fractures (15/27). All Vancouver A fractures were treated with osteosynthesis, while the 2 B1 fractures underwent revision of the femoral stem, one also with osteosynthesis. A total of 13 B2 fractures underwent femoral stem revision with osteosynthesis, one was treated only with osteosynthesis and one only with revision of the stem. The B3 fracture had femoral implant revision and osteosynthesis. Three Vancouver C fractures were treated with osteosynthesis and one with revision of the stem. The osteosynthesis varied from cerclage wiring to plates with screws and/or cerclage wiring. Three patients had measurable LLD clinically and 10 patients had a positive Trendelenburg gait at the follow-up evaluation. Three patients were not available for the Trendelenburg test due to impaired balance. The median ASA score was the same at the time of primary surgery and fracture surgery (Table 3). There were no postoperative infections following the surgery for periprosthetic femoral fracture.

**Table 3.** American Society of Anesthesiology (ASA) score classification at time of surgery.

ASA classification	Primary surgery	Fracture surgery
ASA 1	2	-
ASA 2	20	21
ASA 3	5	6
ASA 4	-	-

## Discussion

Overall, the PROMs after fracture surgery were similar to the PROMs registered the first year following primary THA. Further, the PROMs after both surgeries were improved compared to the preoperative PROM before index surgery. A periprosthetic femoral fracture is a relatively rare complication, and therefore only a limited number of patients were eligible for the present study. However, to our knowledge no study has previously included preoperative PROMs and PROMs the first postoperative year of index surgery, for comparison to PROMs after revision due to a periprosthetic femoral fracture.

Other studies have reported more pain and lower function for patients undergoing surgery due to a periprosthetic femoral fracture.^9,10^ In the present study, when comparing pain after fracture surgery to before primary THA, the pain after fracture surgery was decreased. Several studies have reported an increase in HHS from average 75–80 point at three months after primary THA up to average 86–94 points at one year.^23–28^ In the present study mean HHS was 49 before primary THA, 69 and 83 at 3 and 12 months postoperatively and 74 at the postfracture evaluation. Thus the postfracture HHS results fell after fracture surgery, the results corresponding to “fair” results.

The HOOS-PS score after fracture surgery in the present study was 26 which is a poorer results than the typical sub 20 score of HOOS-PS one year after primary THA as the scale for HOOS-PS is reversed and lower numbers indicates the best results.^29–31^ Winther et al.^31^ reported results for primary THAs from the same institutional registry as used in the present study, a mean HOOS-PS of 22 and 17 at 3 and 12 months follow-up. The mean values for patients in the present study were 30 and 27 at the same time points, indicating that the patient population in this study had worse HOOS-PS results than in other studies. However, the postfracture results for the present study population were actually similar to the results before fracture. The sample size of the present study was not large enough to evaluate if there was a coherence between the risk of periprosthetic femoral fracture and mean HOOS-PS. With PROMs now increasingly being included in national arthroplasty registries, this potential correlation can perhaps be evaluated in a larger data set in the future.

In our study, the mean FJS after fracture treatment was 58 (range 0–100). HOOS-PS and FJS were the functional scores that gave the widest range of variety among the patients. Some patients managed all daily activities, while some needed help with most activities and was sitting in a wheelchair. Only one of the patients included in this study managed running.

A relatively high number of intraoperative fractures were found in the present study. Four of the fractures were detected on postoperative control x-ray, and four fractures were diagnosed within the first four weeks without a new trauma. Most likely, these four patients also had intraoperative fractures, however not detected on postoperative x-ray. The patients had significant pain when weight bearing postoperatively. All eight patients had uncemented stems and seven of the eight patients were women. Uncemented stems are known to increase the risk of intraoperative fracture compared with cemented stems.^3^^32–34^ National guidelines now therefore recommend the use of cemented stems for women aged 75 years and older.^6^ In our study-population, the mean age of the women receiving an uncemented stem at primary surgery was 67 (range, 54–76). Being a teaching hospital, the primary surgeries were performed by both experienced surgeons and those being mentored. This could be one possible explanation of the relatively high number of intraoperative fractures.

Moreta et al.^10^ proposed that Vancouver type A and B3 fractures would experience more pain post fracture surgery due to nonunion after fracture surgery.^10^ In the present study, four out of five patients with type A fractures reported at the post-fracture follow-up a NRS-score 5 or more during activity. Possible explanations for high pain score among these patients might be nonunion, impaired muscle function and hardware irritation.

### Limitations

The most obvious limitation of the study is the number of patients with periprosthetic femoral fractures that were eligible for the study, despite prospectively including patients for more than 10 years. On the other hand, the long inclusion period also meant that 23 patients were deceased at the time for inclusion in the study. This could be due to high age and unrelated causes, but could also represent a selection bias, where these patients had an overweight of fracture-related complications. In addition, 14 patients were not available for the follow-up evaluation due to extensive comorbidity or were simply not reachable and could possibly be a bias for the result. This is a common problem for prospective studies on relatively rare complications such as periprosthetic fractures. The number of patients included is a strong limitation regarding power and statistical analyses, and the results must therefore be interpreted with care. A larger sample size could have opened possibilities for subanalyses of PROMs according to type of fracture, surgical management of the fracture, etc. We still believe that the limited material with inclusion of prefracture PROMs provides new knowledge.

The difference in time from fracture to the follow-up evaluation varied from nine months to 10 years. Studies have reported that the main improvement is observed within the first 6 to 12 months after primary THA and revision surgeries,^35,36^ and remains unchanged for several years thereafter for THA and slowly decreasing for the revision hip arthroplasty patients.^35^ Thus patients with a longer follow-up time could also have inferior results due to ageing and other comorbidities. However, the variation in follow-up time contributes to a more heterogeneous group, possibly affecting PROMs. Another limitation was that only periprosthetic femoral fractures that underwent revision surgery were included. Fractures treated conservatively or discovered and treated intraoperatively were not identified.

### Conclusions

Patients with periprosthetic femoral fractures in the present study had in average less pain than before undergoing primary THA. The pain levels seemed to be comparable with 3 and 12 months after primary THA. Functional outcomes after fracture treatment had a large variation, and was on average reduced to prefracture results.
